# Dissociation and Re-Aggregation of Multicell-Ensheathed Fragments Responsible for Rapid Production of Massive Clumps of *Leptothrix* Sheaths

**DOI:** 10.3390/biology5030032

**Published:** 2016-08-01

**Authors:** Tatsuki Kunoh, Noriyuki Nagaoka, Ian R. McFarlane, Katsunori Tamura, Mohamed Y. El-Naggar, Hitoshi Kunoh, Jun Takada

**Affiliations:** 1 Core Research for Evolutionary Science and Technology (CREST), Japan Science and Technology Agency (JST), Okayama 700-0082, Japan; tkunoh06@cc.okayama-u.ac.jp (T.K.); ktamura@okayama-u.ac.jp (K.T.); hkunoh@cc.okayama-u.ac.jp (H.K.); 2 Graduate School of Natural Science and Technology, Okayama University, Okayama 700-0082, Japan; 3 Advanced Research Center for Oral and Craniofacial Sciences, Okayama University Dental School, Okayama 700-8558, Japan; nagaoka@okayama-u.ac.jp; 4 Department of Physics and Astronomy, University of Southern California, Los Angeles, CA 90089, USA; ian.r.mcfarlane@gmail.com; 5 Molecular and Computational Biology Section, Department of Biological Sciences, University of Southern California, Los Angeles, CA 90089, USA; 6 Department of Chemistry, University of Southern California, Los Angeles, CA 90089, USA

**Keywords:** *Leptothrix*, massive sheath production, sheath fragmentation, bacterial sheath apex, sheath clump, time-lapse microscopy

## Abstract

Species of the Fe/Mn-oxidizing bacteria *Leptothrix* produce tremendous amounts of microtubular, Fe/Mn-encrusted sheaths within a few days in outwells of groundwater that can rapidly clog water systems. To understand this mode of rapid sheath production and define the timescales involved, behaviors of sheath-forming *Leptothrix* sp. strain OUMS1 were examined using time-lapse video at the initial stage of sheath formation. OUMS1 formed clumps of tangled sheaths. Electron microscopy confirmed the presence of a thin layer of bacterial exopolymer fibrils around catenulate cells (corresponding to the immature sheath). In time-lapse videos, numerous sheath filaments that extended from the periphery of sheath clumps repeatedly fragmented at the apex of the same fragment, the fragments then aggregated and again elongated, eventually forming a large sheath clump comprising tangled sheaths within two days. In this study, we found that fast microscopic fragmentation, dissociation, re-aggregation and re-elongation events are the basis of the rapid, massive production of *Leptothrix* sheaths typically observed at macroscopic scales.

## 1. Introduction

Ocherous deposits or floats in neutral waters of lakes, ponds, swamps, drainage ditches, and springs are seen all over the world [1,2,3]. In most cases, these deposits are iron- or manganese-containing materials produced by Fe-/Mn-oxidizing bacteria, biomineralizing organisms that inhabit the groundwater-outwelling hydrosphere. Species of *Leptothrix* are such bacteria, which characteristically produce copious extracellular and microtubular sheaths encrusted with ocherous iron oxyhydroxides [1,2,3]. This sheath formation by *Leptothrix* cells is proposed to protect them from parasites and/or predators [4], to enable the ready absorption of a limited amount of organic nutrients and minerals [3], and to promote attachment of ensheathed cells to solid surfaces, followed by formation of a primary mat consisting of bacterial cells and exudation and sheaths in slowly running water [5,6].

Previous microscopic observations demonstrated that a chain of about 10 cells of *L. ochracea* left their extending sheath at the rate of 1–2 µm/min and then produced a new hyaline sheath on an extension of the mother sheath [5]. Moreover, according to careful successive phase-contrast observations, cells of *Leptothrix cholodnii* divide regardless of their position in the sheath at 0.01–0.04 µm/min, eventually leading to elongation of the sheath at its terminus [7]. In a 10^3^ L pilot water-purifying tank, in which groundwater is circulated, ca. 150 g (dry mass) of *L. ochracea* sheath materials can accumulate within a day after existing ensheathed cells and sheaths are removed by washing (see Figure S1 in the Supplemental Material and [8]). These reports demonstrate the astonishing rate at which cells of *Leptorthrix* species proliferate and produce Fe/Mn-encrusted sheaths. Such massive production of Fe/Mn-encrusted sheaths often causes significant clogging of water distribution systems [2,9], requiring costly disposal [8].

Remedying these problems requires a better understanding of the mechanisms underlying the generation of Fe/Mn-encrusted sheaths. We wondered whether the high rate of sheath production can be ascribed solely to cell proliferation accompanied by sheath elongation. We thus examined the cell behavior of *Leptothrix* sp. strain OUMS1 (hereafter, OUMS1) using differential interference contrast optics and a light microscope (hereafter, DIC), time-lapse imaging, and transmission and scanning microscopy (TEM and SEM, respectively).

## 2. Materials and Methods

### 2.1. Strain, Medium and Culturing

Cells of *Leptothrix* sp. strain OUMS1 (NITE BP-860) [10] recovered from a frozen stock culture were streaked onto silicon-glucose-peptone (SGP, meaning a liquid medium unless otherwise stated) (see Table S1 in the Supplemental Material for its components) agar plates and incubated at 20 °C for 7 days. Single colonies were transferred to 25 mL of SGP and cultured on a rotary shaker (EYELA FMC-1000, Tokyo Rikakikai, Tokyo, Japan) at 20 °C and 70 rpm. After 2–3 days, 1–5 mL of the cell suspension (adjusted to 10 cfu/mL by densitometry (Nanodrop 2000C, Thermo Fisher Scientific, Waltham, MA, USA)) was transferred to 25 mL of SGP and incubated for 2 days. Because this bacterium forms chains and flocs as they multiplied, the present cfu/mL values are only rough estimates of the bacterium population.

### 2.2. Live/Dead (L/D) Staining and Microscopic Observations

Viability of exponentially growing cells was examined using the Live/dead BacLight Bacterial Viability Kit (Life Technologies, Carlsbad, CA, USA) [11]. Briefly, component B (1.67 mM SYTO9 dye, 18.3 mM propidium iodide) was added to the cell suspensions at 1:300 dilution, and the reaction mixture was kept at room temperature for 20 min. DIC and fluorescence microscopic images of stained cells were obtained with the BX51 system microscope (Olympus, Tokyo, Japan) equipped with a U-MWIB3 dichroic mirror unit (460–490 nm excitation 520 nm emission). Although the relevance of this staining method for judging the live/dead states of only a limited number of bacteria might be questioned, we previously established its validity for judging viability of *Leptothrix* species [12].

### 2.3. Scanning and Transmission Electron Microscopy

For SEM, ensheathed cells and sheaths were fixed with 2.5% *v*/*v* glutaraldehyde in 0.1 M cacodylate buffer (pH 7.0) at 4 °C overnight, post-fixed with 1% *w*/*v* OsO_4_ in the same buffer, then washed with ultrapure water (UPW) and dehydrated in a graded ethanol series and 100% *t*-butanol [13]. Samples were then critical point dried and transferred to a copper stub and coated with platinum for observation with an SEM (S-4300, Hitachi, Tokyo, Japan) at 15 kV.

For TEM, specimens collected by centrifugation were fixed with a mixture of 2% glutaraldehyde and 2% *w*/*v* paraformaldehyde in 0.1 M phosphate buffer (pH 7.4) at 4 °C overnight. After washing with the buffer for 30 min, the specimens were embedded in 3% agar in the buffer. Small pieces of the agar block were post-fixed with 2% OsO_4_ for 1.5 h, then washed with the buffer. Then, the specimens were dehydrated in a graded ethanol series and treated with propylene oxide before embedment in Spurr’s resin. Sections (70–80 nm thick) were stained with uranyl acetate and lead solutions and observed with a TEM (JEM-2100, JEOL, Tokyo, Japan) at 200 kV.

### 2.4. Time-Lapse Imaging of Behaviors of Ensheathed Cells and Sheaths

One milliliter of a two-day-old shake culture of OUMS1 cells was transferred to a 35 mm glass base dish (Iwaki, Tokyo, Japan) and covered with a small agarose pad of SGP containing 0.5%–2% agarose (Wako, Osaka, Japan). Covering with the agarose pad effectively held the specimens in the plane of focus. We confirmed that the agarose pad did not seriously interfere with movement of the bacteria and related structures. The dish was then set on the stage of an inverted microscope (IX73, Olympus, Tokyo, Japan) equipped with a multi-axis stage system (Applied Scientific Instrumentation, Eugene, OR, USA) and observed at 10 × 100 magnification. Time-lapse images (0.5 s to 5 min intervals) were acquired and processed using MetaMorph software (version 7.8.12.0, Molecular Devices, Sunnyvale, CA, USA).

## 3. Results and Discussion

### 3.1. Light Microscopic Imaging of Ensheathed Cells

**Figure 1:**
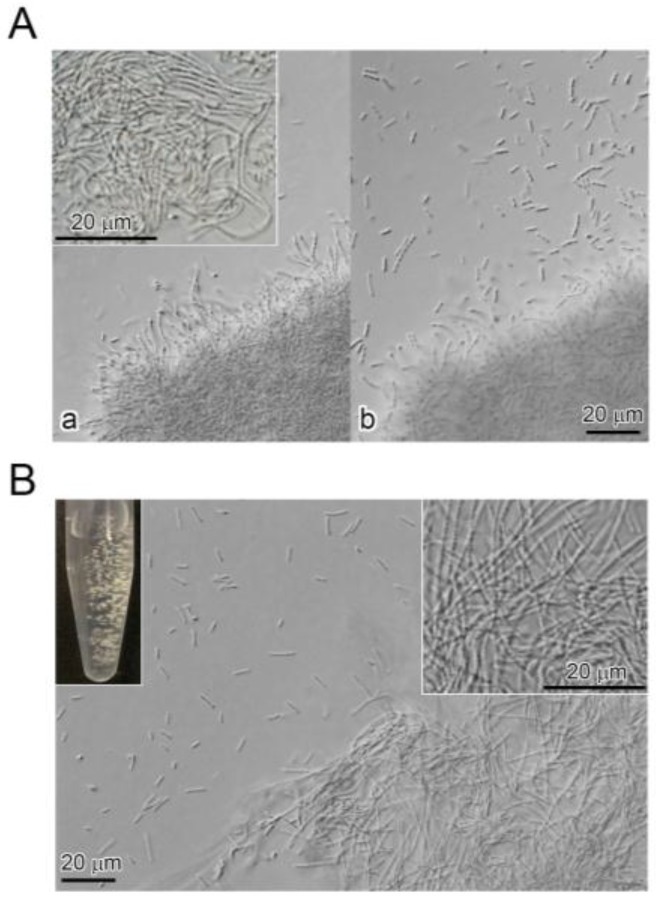
(**A**) colonies of OUMS1 on seven-day-old SGP (silicon-glucose-peptone) agar plate. (**a**) DIC images of the sheath clump comprising tangled immature sheaths (inset); (**b**) numerous motile cells and multicell-ensheathed fragments near the sheath clump; (**B**) visible sheath clumps formed within two days culture (**left** inset) and comprised tangled immature sheaths (**right** inset) and motile cells and multicell-ensheathed fragments outside the sheath clump periphery.

On SGP agar plates, colonies consisted of a raised, fluffy center and a rough, flat margin. In SGP, large clumps of ensheathed cells and sheaths (hereafter referred to as sheath clumps), that comprised loosely tangled sheath-like structures (Figure 1A (a), inset), and numerous single and double/triple chained cells were seen outside the clump periphery by DIC (Figure 1A (b)). Numerous slender, sheath-like filaments extended outward from the clump periphery (Figure 1A (a)). After small pieces of the sheath clump were suspended in SGP and shake-cultured for two days, small, fluffy clumps comprised loosely tangled sheath-looking structures (Figure 1B, right inset), and single and double/triple chained cells were moving outside the clump periphery (Figure 1B).

### 3.2. Electron Microscopic Imaging of Sheath Clumps

**Figure 2:**
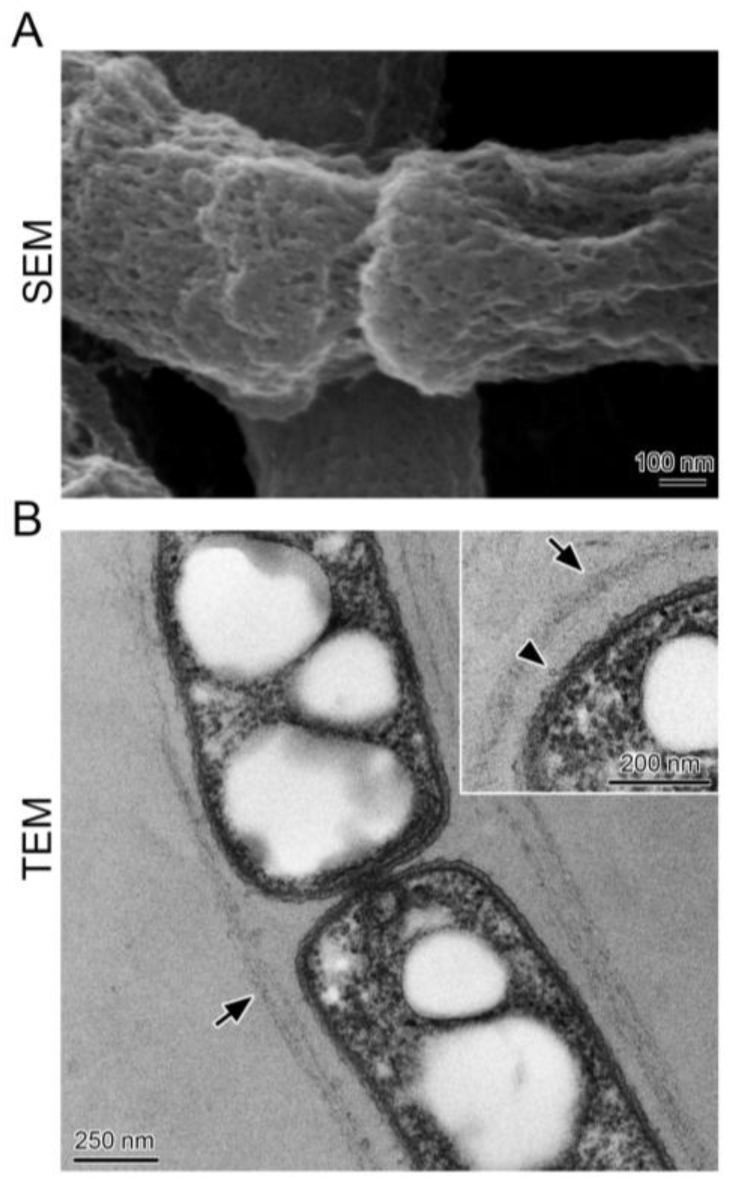
SEM and TEM images of cells encased with the sheath cultured in SGP. (**A**) surface of the cell chain covered with a thin immature sheath of woven fibrils; (**B**) thin layers (corresponding to immature sheaths) (arrows) across an intervening space away from the cells. Inset shows wavy cell surfaces with globular projections (arrowhead) and an immature sheath of woven fibrils.

SEM indicated that surfaces of chained cells were covered with aggregated woven fibrils (Figure 2A), and TEM demonstrated that the chained cells were surrounded by a thin (ca. 50 nm) layer of nearly parallel fibrils (Figure 2B, arrows). A few globular projections (Figure 2B, inset) emerged from the cell surface, consistent with the previous observation in a one-day culture of OUMS1 of a globular and/or thread-like secretion on the surface of the outer membrane of the bacterial cell [14]; these secreted bodies form an immature sheath skeleton comprising aggregated and intermingled fibrils [8]. The surface structure of the sheath in the SEM image appeared to be winkled and shrunk, whereas in the TEM image the surface appeared rather flat. These differing images between the two types of electron microscopy could be due to artifacts created by sample preparation protocols; Dolnalkova et al. [15] emphasized that polymer collapse induced by dehydration could lead to inaccurate spatial relationships and thus affect conclusions regarding the nature of interactions between microbial extracellular polymers and their environment.

Notably, Fe was not detected in this immature sheath by EDX (energy dispersive X-ray microanalyzer) after a one-day incubation even with an Fe source in the medium [14]. Similar to images seen with SEM [16], in culture in SGP lacking Fe, the chained cells were encased with an immature sheath of layered fibrils that were barely distinguishable by light microscopy. Hereafter, the tangled slender structures in the sheath clumps will be called “immature sheaths”, and the chains of several cells moving outside the sheath clump periphery will be called “multicell-ensheathed fragments”.

### 3.3. Time-Lapse Imaging of Behavior of OUMS1 Cells and Multicell-Ensheathed Fragments

**Figure 3:**
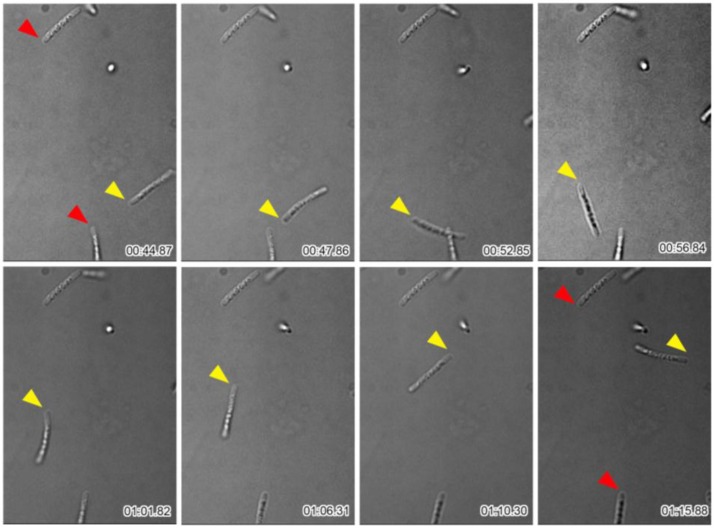
Time-lapse images of multicell-ensheathed fragments found moving around the periphery of the sheath clump. Fragments marked by red arrowheads did not move during the observations; fragment marked by yellow arrowhead moved. Time elapsed is shown at the bottom right of each image.

Many single cells and multicell-ensheathed fragments moved near the periphery of sheath clumps as shown above (Figure 1). To monitor the behavior of these structures in more detail, the cells in SGP were observed with time-lapse at 0.5 s intervals. Although almost half of the cells and the multicell-ensheathed fragments remained at their original positions even at the end of the 4-min observation, others moved rapidly with intermittent short breaks and out of the field of view as summarized (Figure 3). Although OUMS1 cells were reported to have a single polar flagellum [17], the presence of a flagellum within a sheath fragment has not been confirmed, so the driving force of the rapid movement of multicell-ensheathed fragments is an issue to be elucidated.

**Figure 4:**
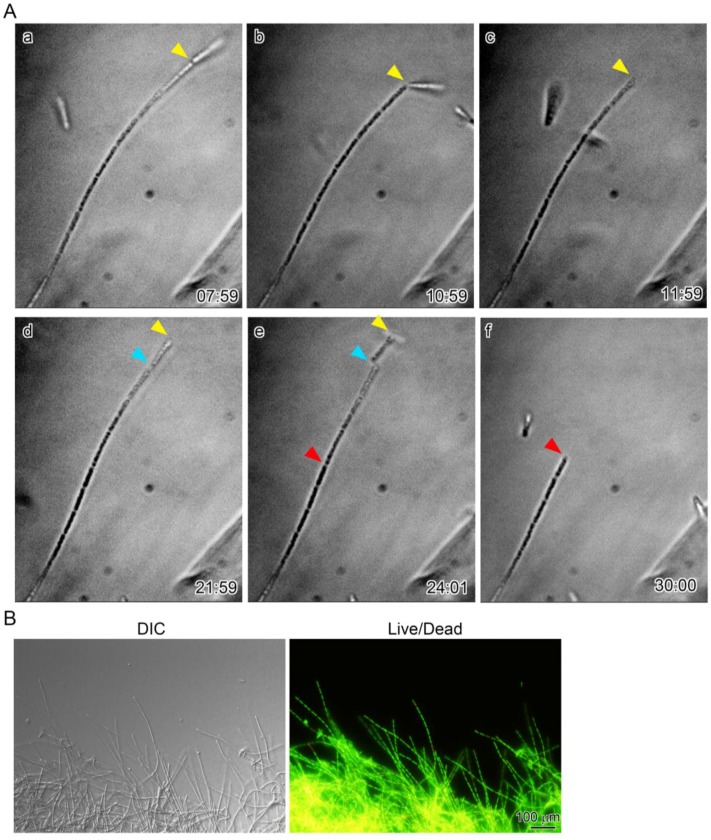
(**A**) time-lapse images showing cleavage of a multicell-ensheathed filament extending from a sheath clump. Yellow, red, and blue arrowheads show, respectively, the first, second, and third cleavages that formed at the apex of the same filament. Three short multicell-ensheathed fragments dissociated from a mother sheath filament within 22 min; (**B**) DIC of a sheath clump with numerous extending immature sheath filaments (**left**) and fluorescent image of the clump, using live/dead staining to show vital cells encased with sheaths (**right**).

According to van Veen et al. [5], shorter sheaths sometimes separated from the main sheath clump of *L. ochracea*, although no time scale for this separation was given. We therefore carefully monitored the multicell-ensheathed fragments at 5-min intervals as they extended from the periphery of a two-day sheath clump (see Video S1 in the Supplemental Material.). Surprisingly, at the apex of the extending sheath filaments, a cleavage appeared (Figure 4A (a), yellow arrowhead) that truncated the apex, thus dissociating a short multicell-ensheathed fragment from the tip of the extending sheath filaments (Figure 4A (b,c)). After 10 min, another cleavage formed at the apex of the same filament (Figure 4A (d), blue arrowhead), dissociating another multicell-ensheathed fragment. Within 2 min, one more cleavage formed midway on the same filament (Figure 4A (e), red arrowhead), dissociating a longer (five cells or more) fragment. Since L/D staining demonstrated that almost all ensheathed cells of the 2-day culture were alive (Figure 4B), this fast, repeated dissociation of the short multicell-ensheathed fragment was not due to cell death. Thus, the majority of the moving fragments near the sheath clump periphery (Figure 1) could be produced by the existing sheath clumps at intervals from approximately 3 to 25 min. Considering that three multicell-ensheathed fragments dissociated from a single extending sheath filament within only 20 min (Figure 4A), an extraordinary number of the fragments could be released from a mass of extending sheath filaments in a single sheath clump within 1 h. As far as we know, these findings are the first that can account for the tremendously massive production of the *Leptothrix* sheaths in natural environments and/or in culture. However, the present study fails to provide numerical data on the sheath fragments. To obtain more persuadable data, we now need to quantify the number of the cells and sheath fragments generated in real time using a technique such as quantitative real-time PCR [18].

At the growing edge of the mat, sheaths were made as *L. ochracea* cells rapidly left behind an Fe oxide encrusted sheath at the trailing end [19]. Therefore, the mode of massive production of sheaths by *L. ochracea* was different from our present results with OUMS1 (closely related to *L. cholodnii* strain SP-6 with 99.9% homology in the 16S ribosomal DNA nucleotide sequence [10]). These results plausibly reflect that the mode of sheath production might differ among the species and/or culture vs. natural environment conditions.

**Figure 5:**
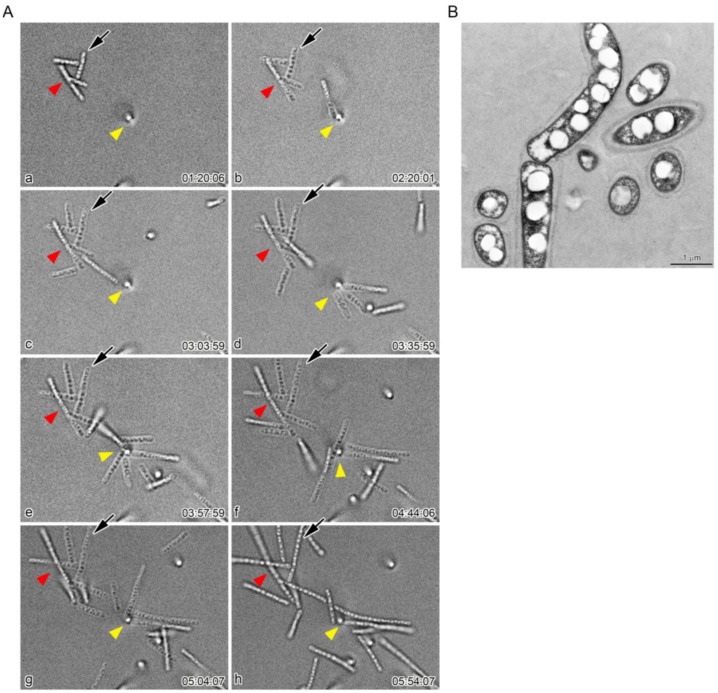
(**A**) time-lapse images of aggregation of multicell-ensheathed fragments. The aggregated fragments increased in number with time (older: red; younger: yellow arrowheads). These aggregated fragments developed into visible sheath clumps within the next 4–5 h. Note that a fragment (arrow) began to elongate again, nearly doubling in length within about 1 h 40 min (frames **b** and **e**); (**B**) TEM image of the fragments covered with sheath layers.

To understand whether these moving multicell-ensheathed fragments were involved in sheath clump formation, we monitored the moving multicell-ensheathed fragments around the sheath clump at 5 min intervals for 20 h (see Video S2 in the Supplemental Material). At a seemingly random time, the motile multicell-ensheathed fragments gradually aggregated to form a clump of fragments (Figure 5A, red and yellow arrowheads) that increased in number within 10–20 min. Once in the clump, the fragments notably began to elongate again (Figure 5, arrows). The number of cells in a fragment increased from six (Figure 5A (b), arrow) to at least 11 (Figure 5A (e)) within 1 h 38 min, amounting to sheath fragment elongation of approximately 0.15 µm/min. Such rapid elongation of multicell-ensheathed fragments plausibly reflects the rapid cell proliferation of *Leptothrix* species. These observations agree with earlier reports on rapid cell proliferation (0.28 µm/min) and sheath elongation (0.01–0.04 µm/min) of *L. ochracea* [5] and *L. cholodnii* [7]. TEM observations confirmed the presence of sheaths around these moving multicell-ensheathed fragments (Figure 5B).

The clumps of multicell-ensheathed fragments enlarged into macroscopically distinguishable sizes within another 4–5 h (Figure 5A), indicating that the aggregation of these moving multicell-ensheathed fragments is an initial step in sheath clump formation by OUMS1. The rapid fragmentation of sheaths, easy aggregation of the multicell-ensheathed fragments, and subsequent recovery of sheath elongation account for the formation of macroscopic fluffy sheath clumps of OUMS1 within two days (Figure 1B). *Sphaerotilus*-*Leptothrix* spp. often attach to solid surfaces [5,6]. In liquid media without solid surfaces, the sheath fragments can attach to each other, thus aggregating and forming large clumps.

To date, many studies [2,9,20,21,22,23] have demonstrated that the sheath matrix of *Leptothrix* is composed of a complicated hybrid of bacterial exopolymers and aquatic metals. The basic skeleton of *L. cholodnii* SP-6, composed of 6.5 nm wide fibrils containing heteropolysaccharides and proteins, contains a relatively high concentration of cysteine residues [20], suggesting that disulfide bonds play an important role in maintaining the structure of the sheath skeleton. Considering that OUMS1 cells cultured in SGP lacking an Fe source are encased with immature sheaths of woven exopolymer fibrils, active groups in the sheath constituent polysaccharides, proteins, and lipids may play an important role in aggregating the multicell-ensheathed fragments to form the sheath clumps. To verify this possibility, we are examining whether chemically blocking active groups will interfere with the aggregation of the multicell-ensheathed fragments.

## 4. Conclusions

The present observations illustrate that the rapid fragmentation of sheaths, ready aggregation of the ensheathed fragments, and subsequent recovery of sheath elongation contribute significantly to the initial stage of sheath clump formation. To our knowledge, this report is the first to account for the mode of rapid massive production of sheaths in *Leptothrix*. However, the modes of cleavage at the sheath apex and the triggering and maintaining factor(s) of the sheath fragment aggregation now need to be elucidated in more detail. In addition, because *Leptothrix* strains usually show a wide morphological range in nature and also under culture conditions, further studies are necessary to examine whether the rapid sheath fragmentation is a common phenomenon for *Leptothrix* spp., especially *L. ochracea* in natural environments. As the first step for these studies, cultivation of OUMS1 under conditions similar to natural outwells is work for the future. Whether the sheath fragmentation is an important step for the morphological life cycle of OUMS1 is another interesting theme to be studied in the near future.

## Supplementary Materials

The following are available online at www.mdpi.com/2079-7737/5/3/32/s1, Figure S1: a pilot water-purifying tank, where groundwater is circulated, is filled with *L. ochracea* sheath materials a day after **r**emoving the existing ensheathed cells and sheaths by washing, Table S1: composition of SGP, Video S1: time-lapse video images of dissociation of the OUMS1 sheath fragments from a single sheath that is extending from the colony edge, Video S2: time-lapse video images of assembly of OUMS1 sheath fragments and elongation of the fragments after the assembly.
