# Supplementary material for: Dissociation and Re-Aggregation of Multicell-Ensheathed Fragments Responsible for Rapid Production of Massive Clumps of Leptothrix Sheaths
Source: Biology (Basel). 2016 Aug 1;5(3):32. doi: 10.3390/biology5030032 (PMC5037351; doi:10.3390/biology5030032)
Supplement: Supplementary file 1 [file biology-05-00032-s001.docx]

Supplemental Materials: Dissociation and
Re-Aggregation of Multicell-Ensheathed Fragments Responsible for Rapid Production of Massive Clumps of *Leptothrix* Sheaths

Tatsuki Kunoh, Noriyuki Nagaoka, Ian R. McFarlane, Katsunori Tamura, Mohamed Y.
El-Naggar, Hitoshi Kunoh and Jun Takada


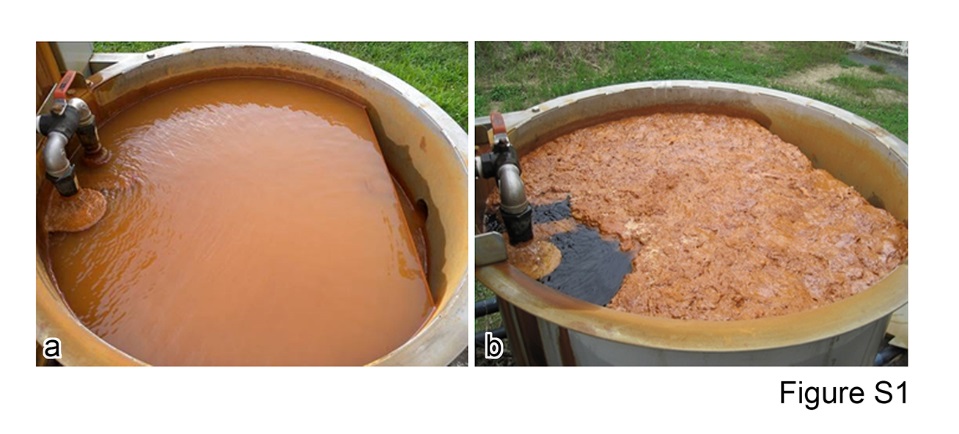


**Figure S1.** (**a**) pilot water-purifying tank (10^3^ L) where outwelling groundwater is circulated (immediately after removing the existing sheath clumps by washing); (**b**) the tank is filled with ca. 150 g (dry mass) of *L. ochracea* sheath materials (1 day after cleaning).

**Table S1.** Composition of silicon-glucose-peptone (SGP).

| **Component** | **Amount (g/L)** | **Concentration (mM)** |
| --- | --- | --- |
| Glucose | 1.000 | 5.55 |
| Soy peptone | 1.000 | ND |
| Na_2_SiO_3_·9H_2_O | 0.200 | 0.70 |
| CaCl_2_·2H_2_O | 0.044 | 0.30 |
| MgSO_4_·7H_2_O | 0.041 | 0.17 |
| Na_2_HPO_4_·12H_2_O | 0.076 | 0.21 |
| KH_2_PO_4_·2H_2_O | 0.020 | 0.15 |
| HEPES | 2.380 | 10.00 |

Medium was adjusted to pH 7.0 with 0.1 N NaOH, then brought to 1 L with ultrapure water.
ND: not determined.

**Video S1.** Time-lapse video images, acquired at 5-min intervals, of dissociation of multicell-ensheathed fragments from a single sheath that is extending from the sheath clump edge (selectively shown in Figure 4). Available at https://www.dropbox.com/s/u6sqhpyiy7r1vuj/Video%202.AVI?dl=0.

**Video S2.** Time-lapse video images, acquired at 5-min intervals, of aggregation of multicell-ensheathed fragments and elongation of fragments after aggregation (selectively shown in Figure 5). Available at https://www.dropbox.com/s/270j0kx1bh4ru0z/Video%203.AVI?dl=0.

© 2016 by the authors; licensee MDPI, Basel, Switzerland. This article is an open access article distributed under the terms and conditions of the Creative Commons by Attribution (CC-BY) license (http://creativecommons.org/licenses/by/4.0/).
